# Aberrant epigenetic silencing of neuronatin is a frequent event in human osteosarcoma

**DOI:** 10.18632/oncotarget.27583

**Published:** 2020-05-19

**Authors:** Haleema Saeed, Sayantani Sinha, Christine Mella, Jeffrey S. Kuerbitz, Monica L. Cales, Mark A. Steele, Jennifer Stanke, Derek Damron, Fayez Safadi, Steven J. Kuerbitz

**Affiliations:** ^1^Division of Pediatric Hematology/Oncology, Akron Childrens Hospital, Akron, OH, USA; ^2^Department of Biological Sciences, Kent State University, Kent, OH, USA; ^3^College of Osteopathic Medicine, University of Pikeville, Pikeville, KY, USA; ^4^Department of Anatomy and Neurobiology, Northeast Ohio Medical University, Rootstown, OH, USA; ^5^Musculoskeletal Research Group, Northeast Ohio Medical University, Rootstown, OH, USA; ^6^Rebecca D. Considine Research Institute, Akron Children’s Hospital, Akron, OH, USA; ^7^Department of Pediatrics, Northeast Ohio Medical University, Rootstown, OH, USA; ^8^Current affiliation: Shaukat Khanum Cancer Hospital and Research Centre, Lahore, Pakistan; ^9^Current affiliation: Department of Hematology, Children’s Hospital of Philadelphia, Philadelphia, PA, USA; ^10^Current affiliation: Division of Developmental Biology, Cincinnati Children's Hospital Medical Center, Cincinnati, OH, USA; ^11^Current affiliation: Penn State Health St. Joseph, Reading, PA, USA; ^12^Current affiliation: Foundation Medicine, Boston, MA, USA

**Keywords:** osteosarcoma, DNA methylation, neuronatin, tumor suppressor genes

## Abstract

The paternally imprinted neuronatin (*NNAT*) gene has been identified as a target of aberrant epigenetic silencing in diverse cancers, but no association with pediatric bone cancers has been reported to date. In screening childhood cancers, we identified aberrant CpG island hypermethylation in a majority of osteosarcoma (OS) samples and in 5 of 6 human OS cell lines studied but not in normal bone-derived tissue samples. CpG island hypermethylation was associated with transcriptional silencing in human OS cells, and silencing was reversible upon treatment with 5-aza-2’-deoxycytidine. Expression of NNAT was detectable in osteoblasts and chondrocytes of human bone, supporting a potential role in bone homeostasis. Enforced expression of NNAT in human OS cells lacking endogenous expression resulted in significant reduction in colony formation and *in vitro* migration compared to nonexpressor control cells. We next analyzed the effect of NNAT expression on intracellular calcium homeostasis and found that was associated with an attenuated decay of calcium levels to baseline following ATP-induced release of calcium from endoplasmic reticulum (ER) stores. Furthermore, NNAT expression was associated with increased cytotoxicity in OS cells from thapsigargin, an inhibitor of calcium reuptake into ER and an inducer of the ER stress response. These results suggest a possible tumor suppressor role for NNAT in human osteosarcoma. Additional study is needed ascertain sensitization to ER stress-associated apoptosis as a mechanism of NNAT-dependent cytotoxicity. In that case, epigenetic modification therapy to effect *NNAT* transcriptional derepression may represent a therapeutic strategy potentially of benefit to a majority of osteosarcoma patients.

## INTRODUCTION

Osteosarcoma (OS) is the most common primary bone tumor in children, adolescents, and young adults. Although OS is a comparatively rare cancer, affecting fewer than 500 individuals below 21 years of age annually in the United States [1], some clinical features are well-documented including a characteristic range of age at presentation, prevalent sites of primary tumor involvement, and typical patterns of metastatic dissemination. Response to chemotherapy, which frequently may be assessed at the time of primary tumor resection following neoadjuvant chemotherapy, is highly variable, however, and much uncertainty persists regarding the molecular events governing the biology of these tumors and response to therapy. Presumed to originate in pluripotent mesenchymal cells, OS typically produce osteoid as a marker of osteoblastic differentiation but may also exhibit features of chondroblastic and fibroblastic development, and histologic heterogeneity of tumors is well documented. Molecular and cytogenetic analyses have indicated a substantial prevalence of structural variation in OS [2–5]. Despite this genomic complexity, and excepting the well-established roles for aberrations in the RB transcriptional corepressor 1 (*RB1*) [6, 7], and tumor protein p53 (*TP53*) [8–10] tumor suppressor pathways in hereditary OS predisposition as well as sporadic tumors, few pathognomonic cytogenetic abnormalities or consistent genetic mutations have emerged either as clear and predominant drivers of tumorigenesis or as biomarkers for histologic features or clinical behavior. Comparatively few studies, furthermore, have explored the potential role of epigenetically-mediated gene dysregulation in the pathogenesis of OS.

Epigenetic mechanisms, including aberrant CpG island hypermethylation leading to transcriptional silencing of gene expression, are now well established as mediators of gene dysregulation determining diverse neoplastic phenotypes in essentially all types of cancer [11–13]. Human neuronatin (*NNAT*) is a paternally imprinted gene (paternally-derived allele is transcriptionally active, maternally derived allele is transcriptionally silent) localized to chromosome segment 20q11.2. We first identified aberrant hypermethylation of the *NNAT* 5′ CpG island childhood acute leukemia [14]. Subsequent analyses have shown *NNAT* hypermethylation in pituitary adenoma [15] and non-small-cell lung cancer (NSCLC) [16]. Enforced expression of *NNAT* has been associated with decreased *in vitro* colony forming efficiency (CFE) and inhibition of proliferation in pituitary adenoma cells [15, 17] and decreased CFE in NSCLC [16], suggesting a potential tumor suppressor function in some cancers.

The *NNAT* gene is expressed as two alternatively spliced mRNA isoforms, both of which encode an endoplasmic reticulum-associated proteolipid [18, 19]. Sequence homology [18] and functional analyses [19–21] have suggested that NNAT acts as a regulator of the sarco/endoplasmic reticulum Ca^2+^ATPase (ATP2A2, SERCA2), thereby participating in the regulation of intracellular Ca^2+^ levels ([Ca^2+^]_i_) in some cells. Originally described as a gene selectively expressed in the developing brain, *NNAT* has indeed been shown to play a role in the induction of neural differentiation in embryonic stem cells via inhibition of SERCA2 [21]. Additional observations, however, suggest a more pleotropic role. *NNAT* expression has been shown to induce adipocytic differentiation in mesenchymal cells [19] and to induce apoptosis in pancreatic cells [20]. The roles ascribed to *NNAT* of induction of differentiation in cells of mesenchymal origin and silencing/suppression of neoplastic phenotypes in various cancers prompted us to explore a potential role in solid tumors in children, especially those of mesenchymal origin.

We screened a panel of pediatric primary solid tumors to determine the prevalence of *NNAT* CpG island hypermethylation. We found that such aberrant methylation was relatively rare among the most common embryonal tumors of childhood, neuroblastoma and Wilms’ tumor. Notably, however, more than two thirds of osteosarcoma (OS) samples demonstrated aberrant hypermethylation of the CpG island encompassing the *NNAT* promoter and exon 1. We therefore studied the role of hypermethylation of *NNAT* promoter region on *NNAT* expression in OS cells and tested the effect of *NNAT* expression on the clonogenic and invasive capacity of OS cells *in vitro*. Our results show that *NNAT* is silenced by aberrant CpG island hypermethylation in human OS. We also show that enforced expression of NNAT inhibits clonogenicity in human OS cells and suppresses *in vitro* transmembrane migration. Induction of NNAT expression in OS cells resulted in attenuated decay of intracellular calcium levels following mobilization from ER stores, and NNAT expression enhanced the cytotoxic effect of thapsagirgin, an inhibitor of SERCA2 and an inducer of endoplasmic reticulum (ER) stress in OS cells. Together, these findings support a possible tumor suppressor function for NNAT in human osteosarcoma. Establishing a potential mechanism related to calcium homeostasis and/or ER stress warrants further investigation, but the present analysis suggests that *NNAT* hypermethylation may represent a potential target for epigenetic modifier therapy in osteosarcoma.

## RESULTS

### The *NNAT* CpG island exhibits frequent aberrant methylation in pediatric bone sarcomas but not in embryonal or CNS tumors

**Figure 1:**
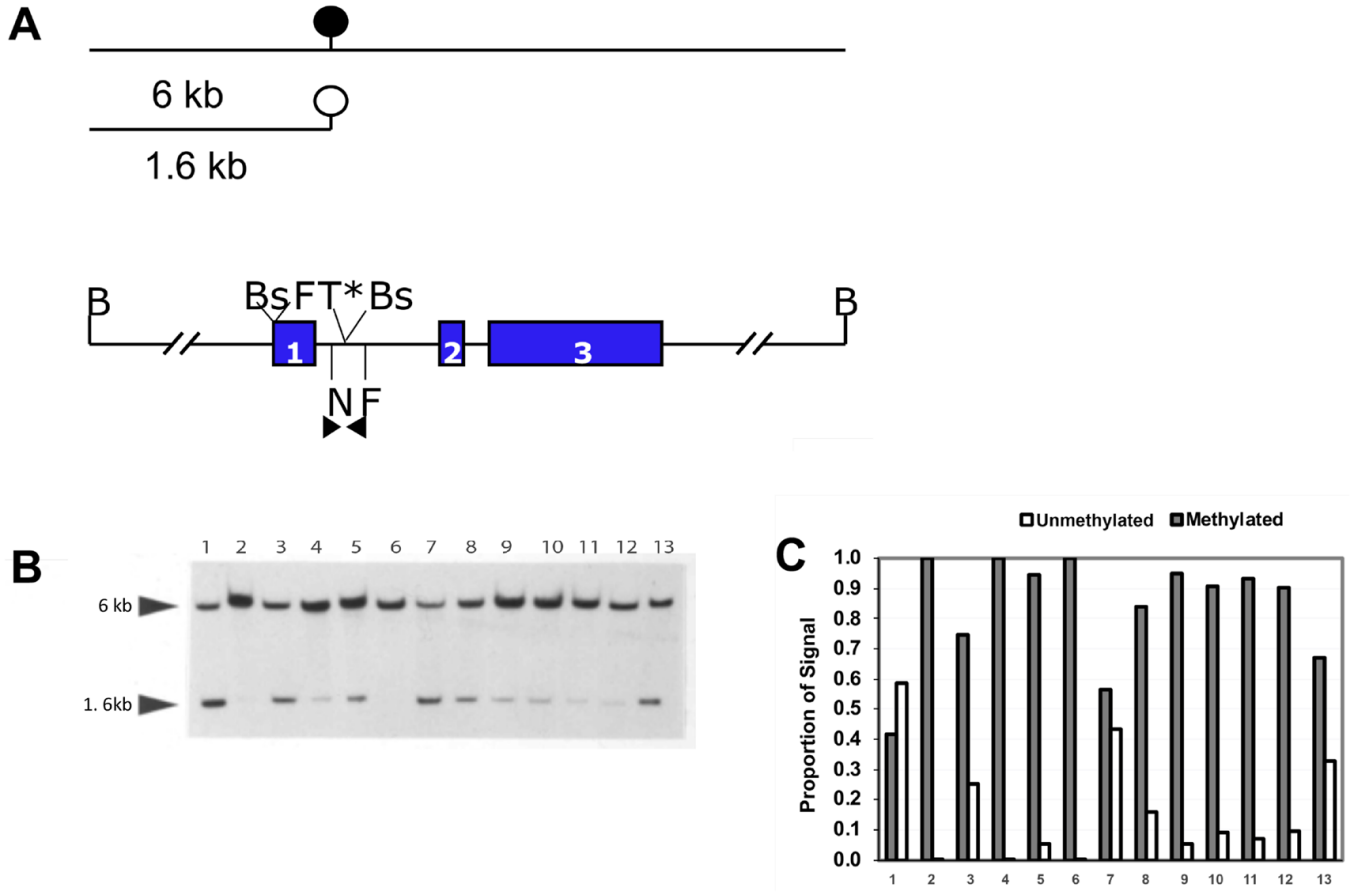
Quantitative methylation analysis shows that the *NNAT* CpG island is hypermethylated in human osteosarcoma tumor samples. (**A**) Schematic and restriction map of *NNAT*. B, BamHI; F, FspI; T, Taq^α^1 (^*^indicates that the site is created by bisulfite modification); N, NruI; Bs, BssHII. Colored rectangles indicate exons. The solid circle indicates a methylated NruI site; the open circle indicates an unmethylated NruI site. Arrowheads indicate the positions and directions of primers for COBRA analysis and bisulfite-mediated PCR. Lines above the restriction map indicate the relative size of restriction fragments produced by digestion of genomic DNA with BamHI and NruI, depending on the methylation status of the NruI site. (**B**) Southern Blot autoradiographic analysis to quantitate NNAT CpG island methylation in human osteosarcoma cell lines and primary tumor samples. Genomic DNA samples were digested with restriction enzymes BamHI and NruI. Following agarose gel electrophoresis and transfer, the membrane was hybridized with a probe generated from the 1.6 kb NNAT upstream BamHI/NruI restriction fragment. DNA samples are: lane 1, cell line HOS; lane 2, cell line MNNG/HOS; lanes 3–13, human osteosarcoma tumor samples. (**C**) Densitometric quantitation of *NNAT* CpG island methylation in human osteosarcoma tumor samples. Bars represent the proportion of total autoradiographic signal representing methylated and unmethylated alleles for each DNA sample.

We have previously shown that hypermethylation of the *NNAT* 5′ CpG island is a frequent event in acute leukemias of childhood [14]. Extending our analysis to common solid tumors of childhood and adolescence, we examined tumor samples by Southern blot utilizing methylation-sensitive restriction endonucleases focusing on the CpG island comprising the promoter, exon 1, and proximal intron 1 (Figure 1A). This tumor panel included Wilms’ tumors, neuroblastomas, OS, Ewing sarcomas, and CNS tumors. Among these neoplasms, we observed that the bone sarcomas OS and Ewing sarcoma exhibited a high frequency of *NNAT* hypermethylation compared to embryonal tumors or CNS tumors (Table 1). In our previous analysis of the *NNAT* CpG island we showed methylated and unmethylated alleles present in approximately equal proportions in normal, mature peripheral blood cells (NNAT non-expressors) and pituitary tissue (NNAT expressor), reflecting the imprinted status, i.e., methylation of the transcriptionally silent maternally-derived allele and lack of methylation of the transcriptionally active paternally-derived allele [22]. For the present analysis, tumor samples exhibiting skewing of the allelic methylation signal ratio to ≥ 75% methylated/≤ 25% unmethylated were defined as hypermethylated because transcriptional silencing was consistently seen at this methylation threshold in pediatric acute leukemia specimens (Kuerbitz, unpublished data). Thus defined, 13 of 68 pediatric solid tumors (19.1%) exhibited *NNAT* hypermethylation (Table 1). Interestingly, of these 13 tumors, 12 were bone-derived tumors. We found that 8 of 11 OS tumors (73%) exhibited *NNAT* CpG island hypermethylation (Figure 1B, lanes 3–13, and Figure 1C) as did 4 of 10 Ewing sarcoma samples. Conversely, hypermethylation of the *NNAT* CpG island was observed in only 1 of 13 Wilms’ tumors, and no *NNAT* hypermethylation was observed among the 14 primary neuroblastoma tumors that we analyzed. Similarly, we examined 20 CNS tumors of various histopathologies including high grade and low-grade gliomas, ependymomas, and medulloblastomas. No *NNAT* hypermethylation was identified among these neoplasms (Table 1).

**Table 1:** Hypermethylation of the NNAT CpG island in pediatric solid tumors

Tumor	*n* Hypermethylated	*n* Analyzed	% Hypermethylated
**Ewing sarcoma**	4	10	40
**Wilms’ tumor**	1	13	7.7
**Neuroblastoma**	0	14	0
**Osteosarcoma**	8	11	72.7
**CNS tumor**	0	20	0
**Total**	13	68	19.1

### The *NNAT* CpG island exhibits aberrant methylation in human OS cell lines and tumor samples but not in normal bone tissues

**Figure 2:**
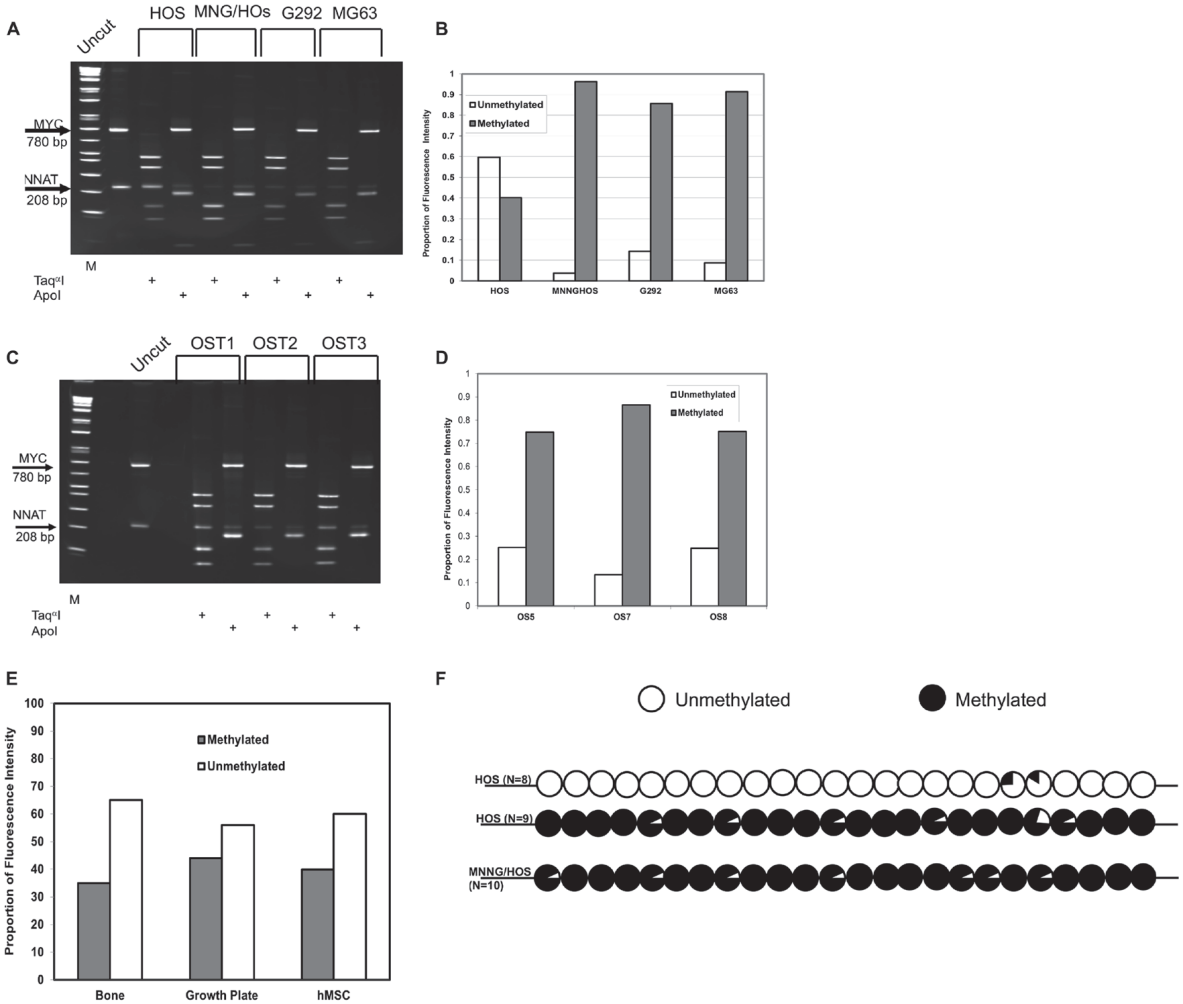
COBRA analysis demonstrates *NNAT* hypermethylation in osteosarcoma cell lines and primary tumor samples but not in normal bone or bone progenitors. (**A**) Genomic DNA from osteosarcoma cell lines was bisulfite modified and PCR-amplified with methylcytosine-specific primers. Products were digested with Taq^α^I to assess methylation or ApoI to assess the completeness of bisulfite modification. A 280 bp PCR fragment from the *MYC* CpG island was added to osteosarcoma DNA amplification products as a Taq^α^I digestion control. Digested DNA was fractionated on a polyacrylamide gel. Restriction fragment lengths are as follows: *MYC*/Taq^α^I – undigested 780 bp, digested – 424 bp and 356 bp; *NNAT*/Taq^α^I – undigested 208 bp, digested 127 bp and 81 bp. DNA samples are (L to R): 1 kb marker, undigested control PCR products, HOS/Taq^α^I, HOS/ApoI, MNNGHOS/Taq^α^I, MNNGHOS/ApoI, G-292/Taq^α^I, G-292/ApoI, MG-63/Taq^α^I, MG-63/ApoI. (**B**) The proportions of methylated versus unmethylated *NNAT* alleles were quantitated for Figure 2A by analysis of the ethidium bromide-stained gel under UV illumination. The proportion of unmethylated *NNAT* alleles equals the proportional intensity of the 208 bp *NNAT*/Taq^α^I band while the proportion of methylated *NNAT* alleles equals the sum of the proportional intensities of the 127 bp plus the 81 bp *NNAT*/Taq^α^I bands. Shown is a representative result of 3 experiments. (**C**) COBRA analysis of *NNAT* allelic methylation in 3 representative osteosarcoma primary tumor samples (OS) was performed as described for Figure 2A. DNA samples are (L to R): 1 kb marker, undigested control PCR products, OS1/Taq^α^I, OS1/ApoI, OS2/Taq^α^I, OS2/ApoI, OS3/Taq^α^I, OS/ApoI. (**D**) Quantitative analysis of the COBRA Figure 2C was performed as described for Figure 2B. Shown is a representative result of 3 experiments. (**E**) Quantitative COBRA methylation analysis was performed for normal human bone, human bone growth plate, and cultured human mesenchymal stem cells. Shown is a representative result of 3 experiments. (**F**) Methylation was determined across the *NNAT* CpG island by bisulfite sequencing in HOS and MNNG/HOS human osteosarcoma cells. Circles represent the composite methylation status of 24 CpG sites compiled from sequence analysis of multiple independent clones for each cell line where unfilled reflects no methylation and fully filled reflects full methylation. N indicates the number of independent clones sequenced for each cell line.

*NNAT* CpG island methylation was assessed in OS cell lines. Southern blot analysis showed that human OS cell line HOS (also called TE-85) exhibited about 60% unmethylated and 40% methylated alleles. Analysis of the HOS-derived, N-methyl-N’-nitro-N-nitrosoguanine-transformed cell line MNNG/HOS [23] however, revealed complete absence of the normal 1.6 kb allele, reflecting acquired methylation or loss of the unmethylated allele (Figure 1A and 1B). Methylation analysis by combined bisulfite PCR restriction analysis (COBRA) confirmed the Southern blot results for HOS and MNNG/HOS cells and demonstrated extensive hypermethylation of the *NNAT* CpG island in the OS cell lines G-292 and MG-63 (Figure 2A and 2B). COBRA analysis of the OS cell lines U-2 OS, and SaOS-2 also revealed extensive *NNAT* CpG island hypermethylation (Supplementary Figure 1A and 1B). Thus, *NNAT* hypermethylation was observed in 5 of the 6 OS cell lines examined.

To expand our analysis, we next employed COBRA to assess 22 additional primary or metastatic OS tumor samples. We first compared proportional allelic methylation quantitated by Southern blot to that of COBRA in 7 OS tumor samples and observed good agreement between the 2 methods with respect to percentage methylation (R^2^ = 0.92, Supplementary Figure 2) and full concordance with respect to scoring for hypermethylation. We identified *NNAT* hypermethylation in 15 of the 22 tumor specimens (68%), thus demonstrating a high prevalence of *de novo* methylation or deletion of the expressed, unmethylated (paternally-derived) allele in this cohort of tumors, also (Figure 2C and 2D).

Detailed descriptions of tumor histology were not available for most cases analyzed, so correlation of *NNAT* hypermethylation with specific OS histologies was not possible in this study. Nevertheless, we noted that tumors exhibiting *NNAT* hypermethylation included 2 that were designated chondroblastic and 1 designated fibroblastic. *NNAT* hypermethylation was identified in 16 of 21 samples (76%) derived from primary tumors and in 6 of 10 samples (60%) from metastatic lesions (lung, soft tissue, and brain). For the remaining 2 OS specimens the source was not designated. Based on this limited analysis, it did not appear that *NNAT* hypermethylation was specific either to a particular OS histology or to primary versus metastatic tumors.

To determine the extent of *NNAT* CpG island methylation in normal and developing bone tissues, we then analyzed genomic DNA samples from normal human bone, non-neoplastic human proximal femoral growth plate tissue derived from a slipped capital femoral epiphysis specimen, and cultured human mesenchymal stem cells. Quantitative methylation analysis by COBRA revealed that methylated alleles comprised 35%, 44%, and 40% of *NNAT* CpG island allelic signal, respectively, in bone, growth plate, and MSC samples (Figure 2E, gel photograph not shown). Thus, our analyses identified aberrant hypermethylation of the *NNAT* CpG island in a total of 23 of 33 (70%) human osteosarcoma samples but not in normal bone, developing bone, or bone progenitor cells. These results suggested that aberrant hypermethylation of the *NNAT* CpG island occurs frequently in human OS tumors.

Since methylation analysis by either Southern blot analysis or COBRA is based on restriction enzyme digestion and thus quantitates methylation at a single CpG dinucleotide, we next sought to determine whether the methylation levels at the CpG sites interrogated by these techniques were representative of methylation more broadly across the *NNAT* CpG island. We therefore performed bisulfite sequencing of HOS and MNNG/HOS-derived DNA to evaluate methylation at each of the 24 CpGs within the 208 base pair COBRA amplicon. Sequencing at least 10 clones, selected at random from cloned, PCR-amplified products of bisulfite-modified DNA, we found that 2 distinct populations of alleles were discernible among HOS-derived clones. One population, comprising 9 of 17 clones, was extensively methylated across the CpG island whereas the remaining 8 clones were nearly devoid of methylation (Figure 2F). This result was consistent with the site-specific allelic methylation levels determined by southern blot and COBRA assays and is compatible with the known imprinted status of the gene in normal cells. By contrast, all clones from MNNG/HOS-derived DNA exhibited methylation at nearly all CpG sites across the *NNAT* CpG island amplicon. Based on these results we concluded that the site-specific methylation levels quantitated by Southern blot and COBRA were representative of methylation broadly across the *NNAT* CpG island.

### Aberrant *NNAT* CpG island methylation is associated with transcriptional repression in OS cell lines

**Figure 3:**
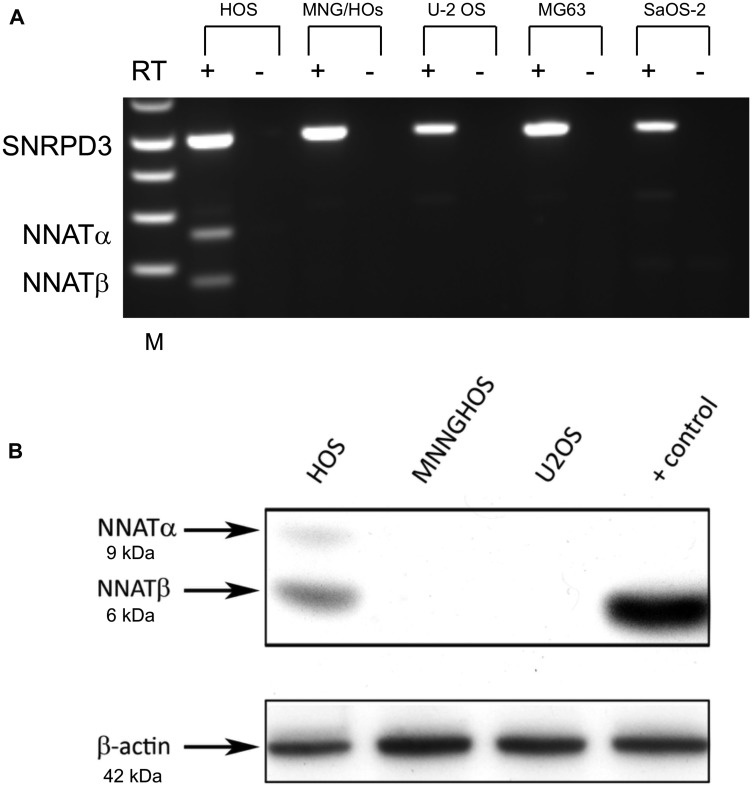
NNAT expression is detected in an osteosarcoma cell line exhibiting the normal imprinted hemimethylation pattern but not in cell lines exhibiting *NNAT* hypermethylation. (**A**) *NNAT* mRNA expression in 5 osteosarcoma cell lines was assessed by semiquantitative endpoint RT-PCR. Total RNA was reverse transcribed and amplified with primers specific for *NNAT* or *SNRPD3* coding sequences. Samples are (L to R): 1 kb ladder, HOS/reverse transcriptase (RT)+, HOS/RT–, MNNGHOS/RT+, MNNGHOS/RT-, U-2 OS/RT+, U-2 OS/RT–, MG-63/RT+, MG-63/RT–, Saos-2/RT+, Saos-2/RT–. The positions of SNRPD3, *NNATα*, and *NNATβ* bands are indicated. (**B**) NNAT protein expression in osteosarcoma cell lines was determined by immunoblot analysis of whole cell lysates. NNAT protein isoforms α and β are indicated. Cell lysate prepared from U-2 OS cells transiently transfected to express NNATβ was used as the positive control. Endogenous β-actin was detected to control for loading.

Our study identified aberrant hypermethylation of the *NNAT* CpG island in 70% of osteosarcoma samples analyzed, indicating that this epigenetic event frequently accompanies osteosarcoma tumorigenesis. In our prior analysis of pediatric acute leukemia we found that *NNAT* CpG island hypermethylation was consistently associated with transcriptional silencing. We therefore analyzed *NNAT* mRNA expression in OS cell lines to determine whether *NNAT* hypermethylation is likewise associated with transcriptional silencing. Semi-quantitative endpoint RT-PCR was performed on total mRNA collected from 5 OS cell lines in log phase growth. *NNAT* mRNA expression was observed only in HOS cells, which exhibited the normal, hemimethylated allelic signal at the *NNAT* CpG island. HOS cells expressed both *NNATα* and *NNATβ* mRNA splice variants. No expression of *NNATα* or *NNATβ* mRNA was evident in any of the remaining 4 OS cell lines, all of which exhibited hypermethylation at the *NNAT* CpG island (Figure 3A). Western blot analysis of whole cell protein lysates prepared from OS cell lines in log phase growth confirmed expression of both NNAT isoforms in hemimethylated HOS cells, but not in the hypermethylated cell lines MNNG/HOS or U-2 OS (Figure 3B). Thus, the normal allelic hemimethylation signature in HOS cells was associated with expression of *NNAT* mRNA and protein isoforms while no *NNAT* expression was detectable in OS cell lines exhibiting aberrant CpG island hypermethylation.

### *NNAT* demethylation is associated with derepressed mRNA expression in OS cell lines

**Figure 4:**
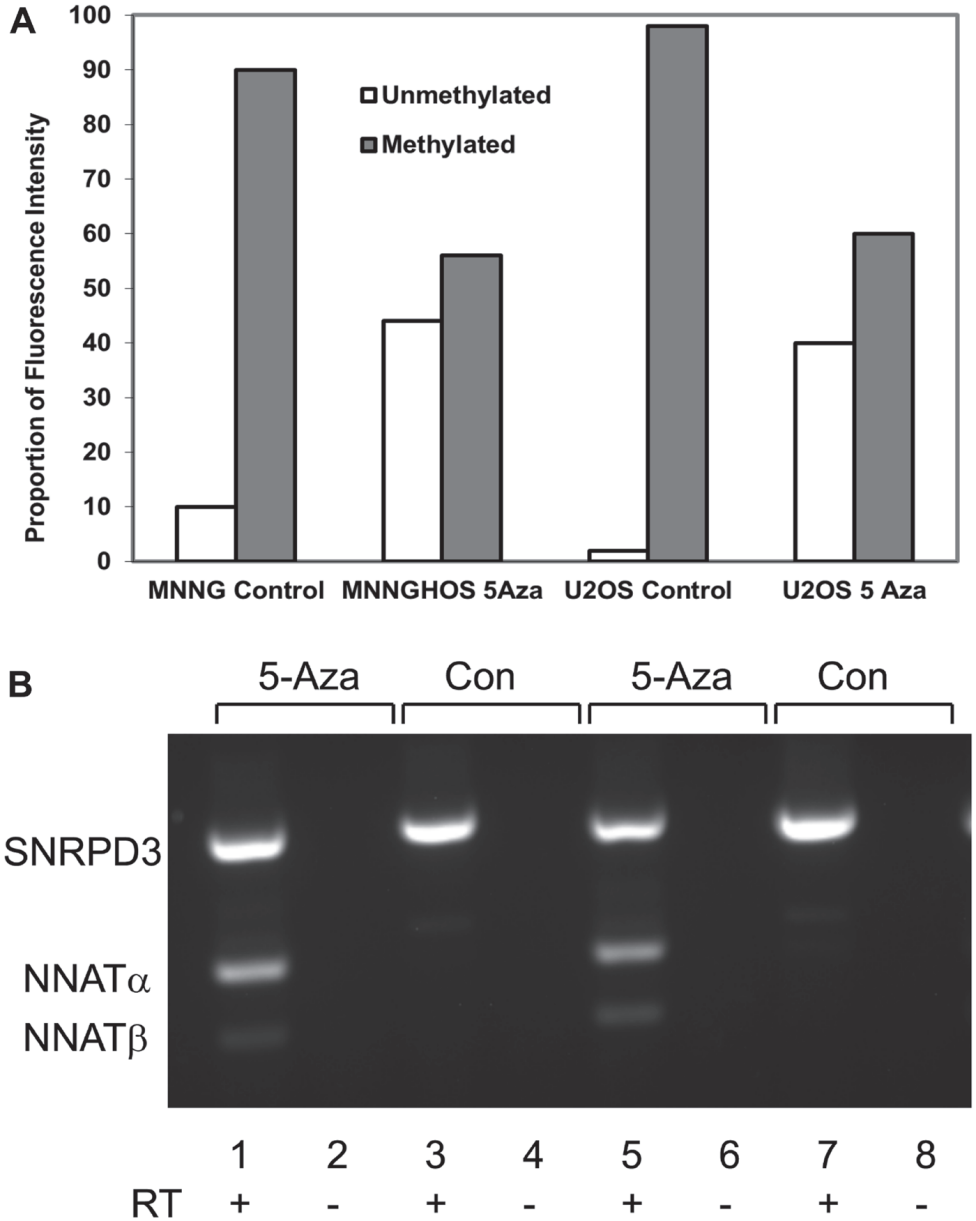
Neuronatin mRNA expression is derepressed with demethylation of the *NNAT* CpG island by 5-aza-2’-deoxycytidine. (**A**) *NNAT* methylation was quantitated by COBRA analysis in genomic DNA from MNNG/HOS cells and U-2 OS cells following treatment for 72 hours with or without 5aza-dC. Quantitative analysis of the ethidium bromide gel image is shown. (**B**) Paired OS cell cultures were grown for 72 hours in medium with 5aza-dC (5-Aza) or without (Con). Total RNA was reverse transcribed (RT) and amplified by semi-quantitative endpoint RT-PCR with primers specific for *NNAT* or *SNRPD3* coding sequences. Shown in the ethidium bromide-stained gel image are PCR products from paired samples treated with or without 5aza-dC and with or without RT: Lanes 1,2,3,4 MNNG/HOS; lanes 5,6,7,8 U-2 OS.

Because relaxation of transcriptional repression with demethylation is a hallmark of epigenetic silencing, we asked whether pharmacologically-mediated demethylation would result in *NNAT* expression in OS cell lines. MNNG/HOS and U-2 OS cells were treated with the DNA methyltransferase inhibitor 5-aza-2’-deoxycytidine (5aza-dC). Both cell lines exhibited *NNAT* hypermethylation and lacked expression of *NNAT* mRNA at baseline. Quantitating *NNAT* CpG island methylation by COBRA, we found that DNA from both cell lines was substantially demethylated after 72 hours of 5aza-dC exposure (≥ 40% unmethylated alleles), while DNA from untreated control cells remained heavily hypermethylated (<10% unmethylated alleles, Figure 4A, gel image not shown). Samples of total mRNA from the 5aza-dC-treated and control cell lines were then analyzed for *NNAT* mRNA expression by semi-quantitative RT-PCR. We found that both *NNAT* splice variants were detectable in the demethylated, 5aza-dC-treated samples, while untreated control samples remained negative for *NNAT* mRNA (Figure 4B). This restoration of *NNAT* mRNA expression in OS cell lines following pharmacologically-mediated demethylation suggested that the absence of baseline expression in these cells was the result of transcriptional repression associated with extensive CpG island hypermethylation.

### NNAT expression is detectable in cells of the bone growth plate

**Figure 5:**
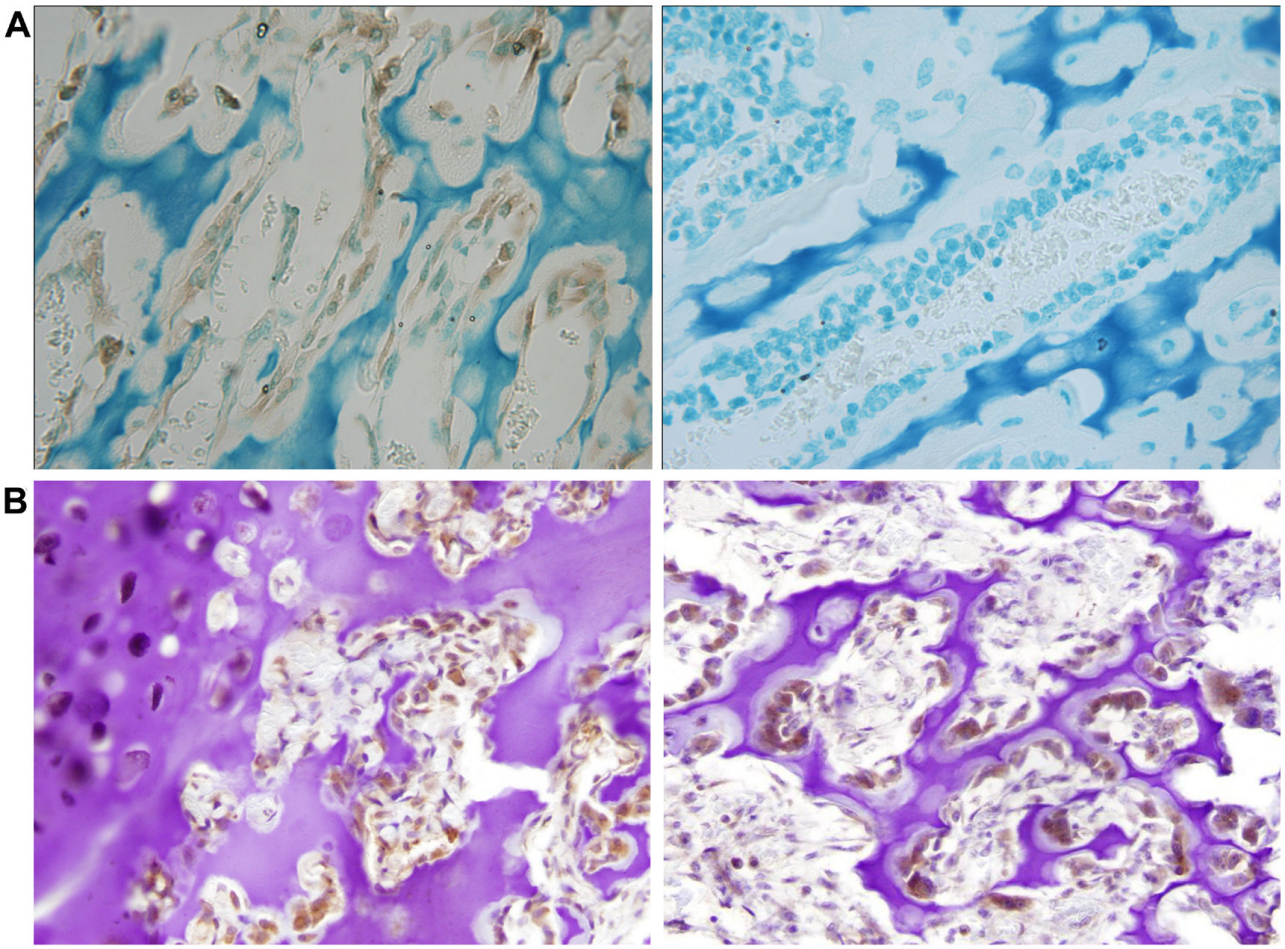
Neuronatin protein expression is detectable in normal bone and cartilage tissues. (**A**) Slides of mouse distal femur growth plate were stained with NNAT antibody and secondary antibody (left) or with secondary antibody alone (right). Magnification 20×. (**B**) Slides of femoral capital growth plate tissue from a 13 year-old human (left) and rib-end growth plate tissue from a 12-week-old human (right) were stained with NNAT antibody and secondary antibody. Magnification 20×.

We reasoned that NNAT expression would likely be detectable in cells during normal bone development if loss of NNAT expression is relevant to the biology of osteosarcoma tumorigenesis. We therefore assessed NNAT expression in normal mouse and human bone growth plate. Utilization of the antibody for identification of NNAT protein by immunohistochemical analysis of human tissue was first validated by testing human anterior pituitary tissue, previously shown to express at high levels. Robust expression was observed in this tissue (Supplementary Figure 3). Mouse distal femoral growth plate sections were then analyzed, and NNAT expression was detected in osteoblasts (Figure 5A). Similarly, NNAT expression was detected in in osteoblasts and chondrocytes from the femoral growth plate of a 13-year-old human (Figure 5B, left panel) and in osteoblasts of human infant rib-end growth plate (Figure 5B, right panel). These results indicated that NNAT was expressed in mesenchyme-derived cells during the course of normal endochondral bone growth in humans and mice.

### Enforced expression of NNAT in OS cells suppresses colony formation and transmembrane migration but does not inhibit cell proliferation

**Figure 6:**
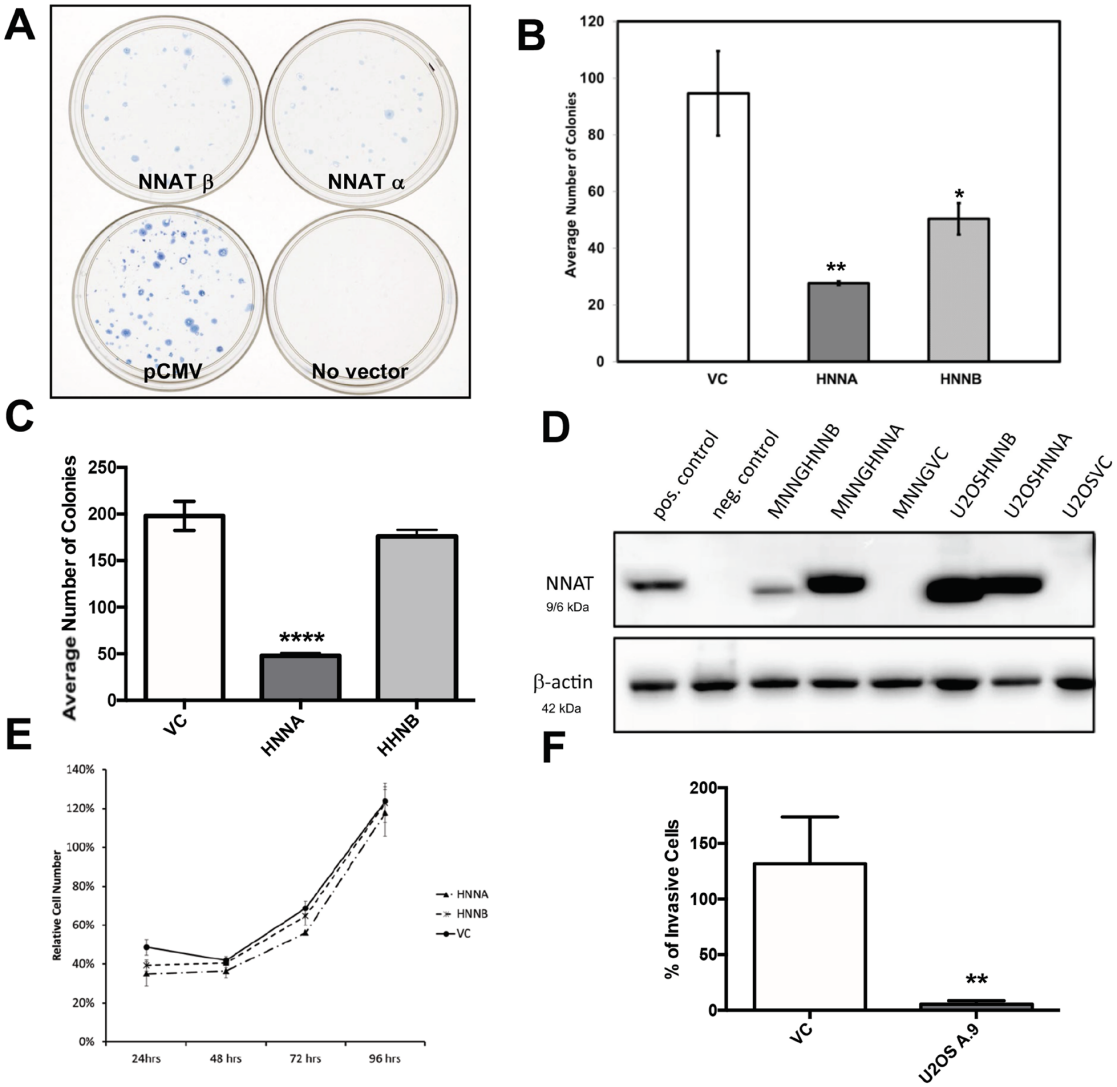
Enforced expression of NNAT in human osteosarcoma cells inhibits clonogenicity and migration, but does not slow proliferation. (**A**) MNNG/HOS cells were transfected with expression vectors encoding NNAT and equal numbers of viable, transfected cells were plated and cultured under antibiotic selection. Colonies were fixed and stained in the culture dish when macroscopically apparent. Shown are dishes of cells transfected to express NNATα, NNATβ, empty vector control (pCMV) or no vector DNA. The result shown is representative of 3 replicate experiments. (**B**) Quantitation of colony formation by MNNG/HOS cells transfected with human NNAT as described. Shown are results for Vector Control, NNATα (HNNA), and NNATβ (HNNB) in a representative experiment of 3 replicates. (**C**) Quantitation of colony formation by U-2 OS cells transfected with human NNAT as described. Shown are results for Vector Control (VC), NNATα (HNNA), and NNATβ (HNNB) in a representative experiment of 3 replicates. (**D**) Human osteosarcoma cells were transfected with expression vectors encoding NNATα or NNATβ. Stable cell lines were isolated and screened by immunoblot analysis of whole cell lysates for NNAT expression. Parent cell lines are MNNG/HOS and U-2 OS. HNNA and HNNB indicate expression of human NNATα and human NNATβ, respectively. VC indicates empty vector control. The positive control is a lysate of MNNG/HOS cells transiently transfected to express NNATα. The negative control is of untransfected MNNG/HOS cells. Expression of β-actin was detected as a loading control. (**E**) Proliferation of MNNG/HOS cells stably transfected with vectors encoding NNATα (HNNA), NNATβ (HNNB), or with empty vector (VC). Equal numbers of cells in log phase growth were seeded in 96 well plates and cell proliferation was quantitated daily by colorimetric assay. Cell numbers for each time point are shown as a percentage of untransfected control cells. (**F**) Migration through Matrigel^®^-coated membranes was assayed in U-2 OS cells stably expressing NNATα (U2OS A.9) versus vector-only non-expressors. Migration from a serum-depleted environment to a serum-replete environment was quantitated by staining membranes and counting cells by visual inspection. Shown is a representative experiment of 3 replicates.

The recurrent methylation-associated loss of expression observed in human OS cells suggested a possible tumor suppressor role for NNAT [15]. We therefore compared colony forming efficiency in OS cells constitutively expressing NNAT versus non-expressor controls. U-2 OS cells and MNNG/HOS cells were transfected with expression vectors encoding either *NNATα* or *NNATβ* or with an empty vector lacking the *NNAT* cDNA. Cells were seeded and grown under antibiotic selection. When macroscopic colonies were counted, a significant reduction in colony number was observed in MNNG/HOS transfected with either *NNATα* or *NNATβ* compared to the vector-only control (Figure 6A, 6B). A similar decrease in clonogenicity was observed in U-2 OS cells transfected with *NNATα*, while colony formation in cells transfected with *NNATβ* was not statistically different from that of vector-only control transfectants (Figure 6C). In parallel experiments, transfectant colonies were also selected and expanded to derive stable cell lines expressing *NNATα* or *NNATβ*. NNAT expression was confirmed in these cells lines by western blot analysis (Figure 6D). Cell proliferation during log phase growth was then compared between MNNG/HOS-derived stable NNAT expressor cells and vector only controls. No difference was observed in proliferative rate between cells expressing NNATα or NNATβ or empty-vector non-expressors compared to untransfected control cells (Figure 6E). These experiments indicated that enforced NNATα expression inhibited colony formation in transfected OS cells while NNATβ did so in a cell line-specific way. However, expression of NNAT isoforms did not slow proliferation of OS cell lines, once established.

### NNAT expression suppresses transmembrane migration potential in U-2 OS cells

To gauge the potential effect of NNAT expression on U-2 OS cells’ capacity for invasiveness, we examined the *in vitro* migration of NNAT-expressor cells versus empty vector controls across Matrigel^®^-coated membranes, a surrogate for invasive potential. Using serum-depleted and serum-replete environments to provide a transmembrane gradient to promote cell migration, we observed decreased transmembrane cell migration in the NNATα-expressor cell line compared to the control (Figure 6F).

### NNAT expression is associated with an attenuated decay of [Ca^2+^]_i_ levels following calcium release

**Figure 7:**
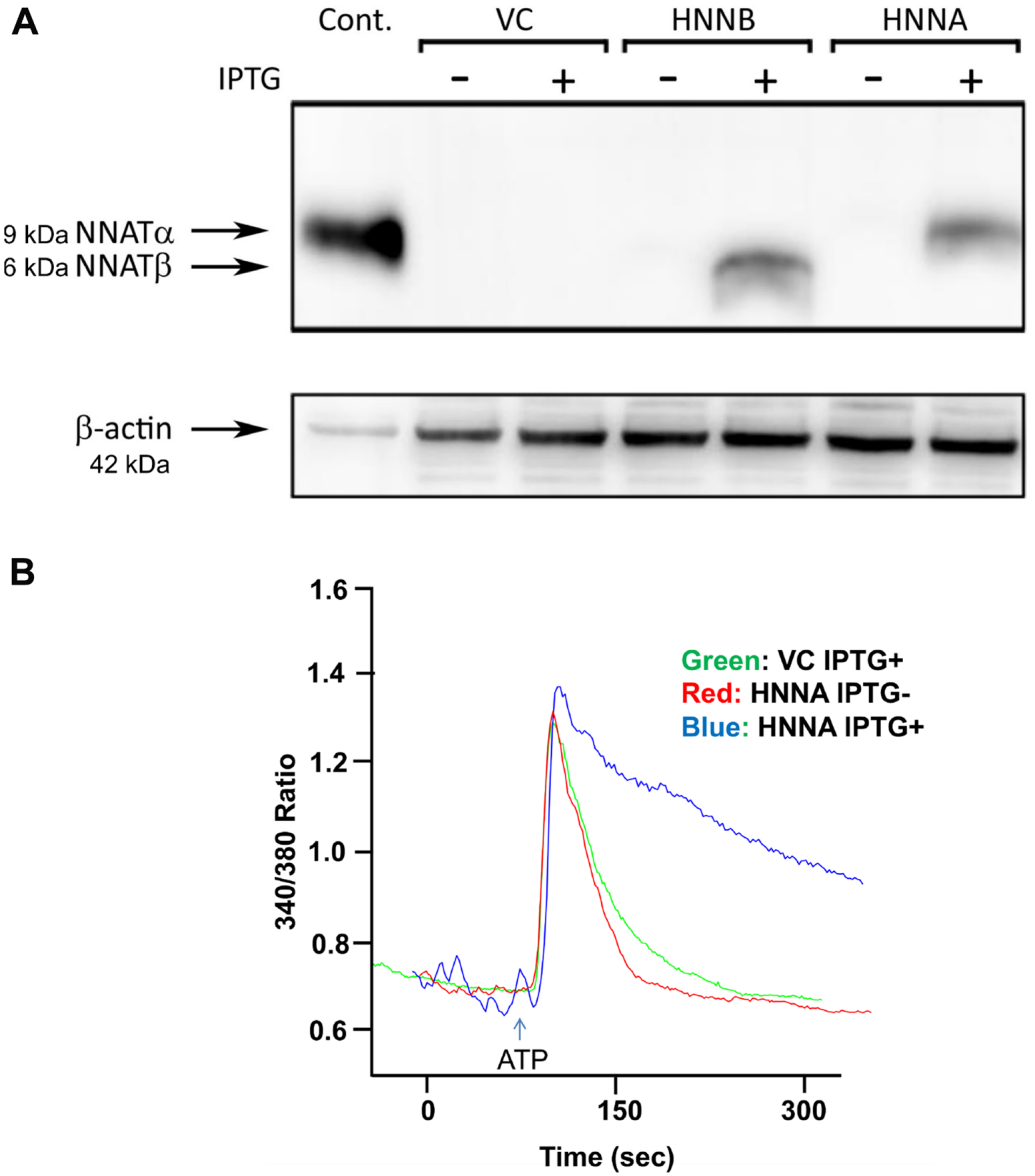
NNAT expression is associated with attenuated decay of [Ca^2+^]_i_ following ATP-induced release of calcium stores in human osteosarcoma cells. (**A**) MNNG/HOS cells were engineered for stable, IPTG-inducible expression of NNATα or NNATβ as described in Methods. NNATα (HNNA) and NNATβ (HNNB) expression was assayed by immunoblot in whole cell lysates of cells with and without IPTG treatment as indicated. VC represents empty vector control non-expressors. A lysate of MNNG/HOS cells transiently transfected to express NNATα was used as a positive control. (**B**) [Ca^2+^]_i_ was measured by fluorescence imaging in fura-2/AM-loaded cells following ATP stimulation. HNNA cells were imaged with (blue tracing) and without IPTG (red tracing) induction of NNATα expression. VC cells are empty vector-control non-expressors imaged after IPTG treatment (green tracing). Shown are representative tracings from 3 replicate experiments.

Expression of NNAT has been associated with increased intracellular calcium levels ([Ca^2+^]_i_) in previous studies, suggesting that NNAT may play a role in regulation of calcium homeostasis in human cells [19–21]. We therefore compared [Ca^2+^]_i_ in human OS NNAT expressors versus nonexpressors. To avoid inter-cell line variability in calcium response, we engineered human MNNG/HOS osteosarcoma cells for inducible NNAT expression. Robust induction of NNAT expression was achieved using the LacSwitch II vector system (Agilent). Using this system, NNAT transcription, blocked at baseline by a constitutively-expressed lac repressor, was derepressed by treating cell cultures with isopropyl β-D-1-thiogalactopyranoside (IPTG). Tight induction of NNAT expression with IPTG treatment, with little or no detectable NNAT in IPTG-untreated cells, was confirmed by western blot analysis (Figure 7A). [Ca^2+^]_i_ was then tracked in individual cells following ATP-stimulated mobilization of calcium stores for NNAT expressor cells with and without IPTG-induction and in empty vector control cells treated with IPTG. In analysis of multiple cell tracings, no difference in the dynamics of [Ca^2+^]_i_ increase—i.e., the rate of [Ca^2+^]_i_ increase or peak [Ca^2+^]_i_—was observed associated with IPTG-induced NNATα expression (Figure 7B). However, the decay of [Ca^2+^]_i_ from the post-release peak throughout the decline back to baseline was markedly attenuated in cells with IPTG-induction of NNATα compared to uninduced cells or IPTG-treated vector-control nonexpressor cells. The [Ca^2+^]_i_ tracing following ATP stimulation of IPTG-untreated NNATα cells (i.e., lacking induced NNATα expression) was not appreciably different from those of the IPTG-treated empty vector control cell line, suggesting that the IPTG itself had little or no effect on the dynamics of Ca_i_ release and re-uptake. A similar attenuation of [Ca^2+^]_i_ decay was observed in MNNG/HOS cells engineered for IPTG-inducible NNATβ expression (Supplementary Figure 4). Together these data suggested that NNAT expression may have inhibited reuptake of Ca^2+^ into intracellular stores or otherwise delayed the return of [Ca^2+^]_i_ to baseline after Ca^2+^ release. The results provided further support for a role for NNAT in regulation of Ca^2+^_i_ homeostasis.

### NNAT expression enhances thapsigargin cytotoxicity in OS cells

**Figure 8:**
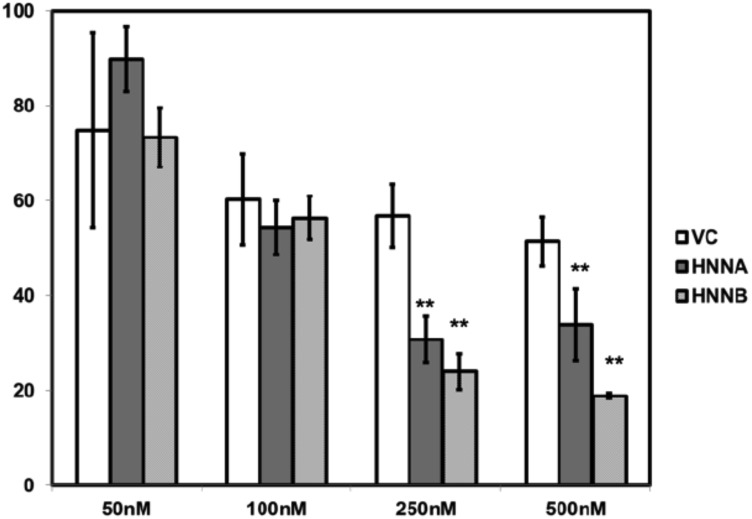
Neuronatin expression enhances thapsagargin cytotoxicity in human osteosarcoma cells. MNNG/HOS cells constitutively expressing either NNATα (HNNA), NNATβ (HNNB), or empty vector nonexpressor cells (VC) were seeded in 96 well plates and cultured for 72 hours in medium with thapsagargin in concentrations of as shown. Cell survival was quantitated by colorimetric assay.

Ca^2+^_i_ homeostasis is critical to normal processing of nascent proteins in the ER, and disruption of Ca^2+^_i_ homeostasis can trigger the ER stress response [24]. We therefore asked whether NNAT expression could affect cell cytotoxicity resulting from ER stress induced by thapsagargin. Thapsigargin is an inhibitor of SERCA2b and induces ER stress associated with elevation of [Ca^2+^]_i_. Prolonged exposure to this agent triggers apoptosis in many cell types. We therefore assessed cell survival in NNATα or NNATβ expressor OS cells treated with thapsigargin compared to control nonexpressors. We found that MNNG/HOS cells constitutively expressing either NNATα or NNATβ exhibited additive cytotoxicity (decreased cell survival) with thapsigargin treatment at 250 nM or 500 nM at 72 hours in compared to nonexpressor controls cells (Figure 8). This result suggested that NNAT expression augmented ER stress-associated cytotoxicity induced by thapsigargin.

## DISCUSSION

We first reported a potential role for epigenetic dysregulation of the *NNAT* gene in human oncogenesis, identifying aberrant 5′CpG island hypermethylation associated with transcriptional silencing in pediatric acute leukemias [14]. The present study was undertaken when a preliminary screen of childhood solid tumors identified frequent aberrant *NNAT* hypermethylation in OS, in contrast to embryonal tumors such as neuroblastoma and Wilms’ tumor or CNS tumors (Table 1). Whereas the normal human bone and cultured primary human mesenchymal stem cell samples we examined exhibited the hemimethylated pattern that is expected at an imprinted gene, we found extensive aberrant hypermethylation of the *NNAT* CpG island promoter region in 23 of 33 primary OS samples and in 5 of the 6 OS cell lines analyzed. Importantly, while restriction digestion-based methods such as southern blot analysis and COBRA typically interrogate single CpG dinucleotides, bisulfite sequencing of OS cell line DNA confirmed that the quantitative methylation levels indicated by these site-specific methods were representative of allelic methylation levels across the *NNAT* CpG island.

DNA methylation-associated, allele-specific expression or transcriptional repression is well established in genes subject to imprinting or to X-chromosome inactivation in normal cells [25]. Similarly, transcriptional silencing of tumor suppressor genes resulting from aberrant CpG island hypermethylation may be regarded as the functional equivalent of mutation-mediated loss of activity in cancers [12]. While we identified *NNAT* hypermethylation in OS employing a dedicated screen of childhood solid tumors, aberrant *NNAT* silencing has also been identified in human pituitary adenomas [15] and in human non-small-cell lung cancers [16] where *NNAT* transcriptional derepression was identified in mRNA expression screens employing RNAi-mediated or pharmacologically-mediated demethylation. In agreement with these reports, we found that extensive *NNAT* CpG island methylation was associated with transcriptional repression and absence of protein expression in 4 of 5 OS cell lines tested. Expression of the 2 mRNA splice variants and 2 protein isoforms was evident only in HOS cells, and these cells exhibited the normal proportional (~50%) allelic methylation ratio.

Because the epigenetic mark we interrogated for *NNAT* silencing is DNA methylation, we ascertained that *NNAT* derepression could be accomplished with CpG island demethylation. We treated OS cell lines with 5aza-dC, a well-characterized and widely-used demethylating agent [26–28]. Important to this analysis, 5aza-dC, a nucleoside analogue that is specific for DNA, acts via inhibition of DNA methyltransferase and does not inhibit RNA or protein synthesis [29]. The resultant loss of *NNAT* CpG island methylation and concomitant gain of *NNAT* mRNA and protein expression observed in the 5aza-dC-treated OS cell lines confirmed the epigenetic basis of *NNAT* silencing in OS cells. Further study to optimize *NNAT* derepression for therapeutic application may utilize, additionally or alternatively, epigenetic modifiers targeting histone modifications or other epigenetic marks.

To assess a potential tumor suppressor role for *NNAT* we tested colony formation in OS cell lines expressing NNAT isoforms. We found that enforced expression of either the α or the β NNAT isoform was associated with significant suppression of clonogenicity compared to empty vector controls in MNNG/HOS cells. This result is in agreement with data reported by Zhong in human non-small-cell lung cancer [16] and Revill in murine pituitary adenoma cells [15]. Significant suppression of clonogenicity was not observed with expression of NNATβ in U-2 OS cells, indicating that this phenotype may be cell line-specific. In contrast to the reported observations in pituitary adenoma cells [17], inhibition of proliferation did not accompany NNAT expression in our analysis. This discrepancy may be related to different experimental methods. Whereas Dudley *et al.* tested assayed proliferation in cells engineered for inducible NNAT expression, cells stably transfected for constitutive NNAT expression were utilized for proliferation experiments in the present study. In the constitutive expression system, proliferative capacity is necessarily established as a function of clone selection. Suppression of clonogenicty was thus more likely the result of inhibition of early events in *in vitro* colony formation rather than expansion of established micro-colonies in the OS cells we analyzed.

When we assessed transmembrane migration, a commonly-employed surrogate for invasive potential, we observed decreased migration in NNAT expressors compared to controls, suggesting possible suppression of invasive potential associated with NNAT expression in OS. Renner and colleagues also evaluated NNAT-dependent cell migration in an *in vitro* wound healing assay and noted decreased migration in liposarcoma cells transfected with *NNATα* [30]. These results showing decreased cellular migration with NNAT expression contrast with results reported by Ryu et al. in which miRNA-708, a suppressor of metastasis in human breast cancer cells, was found to downregulate NNAT expression [31]. In this study, the metastatic phenotype was rescued from miRNA-708-dependent suppression by enforced expression of a *NNAT* mRNA that was rendered refractory to miRNA-708 downregulation by mutation of the 3′UTR. These disparate results suggest that the effect of NNAT expression vis-à-vis metastasis is also tumor-specific or even cell-specific.

Other investigators have demonstrated an effect of NNAT expression on modulation of cellular [Ca^2+^] [18–20]. Tracking [Ca^2+^]_i_ at the single cell level we found that NNAT expression attenuated the decline back to baseline following the release of stores by ATP stimulation. A role for NNAT in intracellular Ca^2+^ homeostasis is suggested by several lines of evidence. *NNAT* encodes a proteolipid and shares protein sequence homology with phospholamban, an inhibitor of SERCA2, the cardiac sarcoplasmic reticulum Ca^2+^ ATPase, type 2 [18, 32]. Immunofluorescence experiments have suggested that NNAT localizes to the ER membrane, the major cellular repository of Ca^2+^ [19, 20]. Physical interaction between NNAT and SERCA2 in embryonic stem (ES) cells was suggested by co-immunoprecipitation experiments reported by Lin *et al*. who found that NNAT expression in ES cells mediated induction of neural differentiation in a Ca^2+^-dependent way [21]. Our observation that NNAT expression did not alter the rate or peak level of [Ca^2+^]_i_ increase in OS cells upon ATP-stimulated Ca ^2+^ release but rather slowed the return to baseline [Ca^2+^]_i_ levels is conceptually consistent with inhibition by NNAT of SERCA2-dependent sequestration of Ca^2+^ from the cytoplasm back into the ER.

We found that NNAT expression in OS cells also augmented cytotoxicity mediated by thapsigargin, an inhibitor of SERCA that induces apoptosis via the ER stress response [33]. This effect on cellular sequelae following perturbation of [Ca^2+^]_i_ further supports, albeit indirectly, an action for NNAT relating to SERCA activity and [Ca^2+^]_i_ homeostasis and supports previous data showing that expression of NNATβ induced expression of ER stress-related proteins and triggered ER-stress mediated apoptosis in pancreatic beta cell-derived cells [20]. A recent report has suggested that NNAT-associated dysregulation of [Ca^2+^]_i_ and associated ER stress underlies the neuropathology in Lafora disease [34].

NNAT expression has been assessed in relation to multiple tumor types, and a consistent picture has not emerged to date. In addition to the present study focused on osteosarcoma, methylation-associated silencing of NNAT together with expression-associated suppression of neoplastic phenotypes such as colony formation, proliferation, or cell migration has been reported in liposarcoma [16] and pituitary adenoma [15, 17]. Together, these reports support a tumor suppressor role for *NNAT*. Because we did not identify tumors exhibiting NNAT promoter hypermethylation among neuroblastomas we examined, we did not evaluate NNAT expression in these tumors. Tajiri and colleagues found, however, that lower level NNAT expression correlated with an unfavorable prognosis in neuroblastoma [35], consistent again with a more aggressive phenotype in tumors lacking NNAT expression. Ryu *et al.* [31] suggest the converse - that expression of NNAT is a driver, not a suppressor, of neoplastic phenotypes. Uchihara *et al.*, in their study of NSCLC, correlated NNAT expression with an unfavorable prognosis [36], in apparent contradiction to the tumor suppressor role for NNAT suggested by Zhong and colleagues in that disease [16]. Xu and colleagues found that NNAT overexpression was associated with shortened survival in patients with glioblastoma multiforme [37]. Similarly, overexpression of NNAT has been noted in medulloblastoma, and enforced NNAT expression, and particularly co-expression of both NNAT isoforms, in a medulloblastoma cell line resulted in increased clonogenicity in soft agar and in tumor xenograft formation [38]. This apparent conflict between promotion and suppression of neoplastic phenotypes may be reconcilable conceptually by noting the consistent finding in recent studies, including the present analysis, of the effect of NNAT expression on [Ca^2+^]_i_. If NNAT functions as an inhibitor of SERCA2 so that silencing or upregulation of NNAT expression results in transient or sustained changes in [Ca^2+^]_i_, then the phenotype resulting from NNAT silencing may be variable among tumor types dependent upon the dynamics of the Ca^2+^_i_ response, the lineage-specific or differentiation stage-specific portfolio of calcium-responsive genes and proteins, and/or the cell sensitivity to ER stress responses including apoptosis resulting from perturbation of Ca^2+^_i_ homeostasis. Detailed analysis of the events downstream of Ca^2+^ release and the effect of NNAT expression, or lack thereof, on these cell-specific processes or properties will be necessary to facilitate a more mechanistic understanding of the roles of NNAT in tumor promotion versus tumor suppression.

The present analysis suggests a tumor suppressor role for NNAT expression in human OS, potentially resulting from sensitization to ER stress-associated cytotoxicity. If confirmed mechanistically, NNAT silencing may represent a potential target for therapy utilizing epigenetic modifiers to achieve derepression. This potential utility of demethylation therapy with decitabine (5aza-dC) to reverse estrogen receptor silencing and inhibit neoplastic phenotypes in OS cells has been demonstrated recently [39]. Compiling a panel of such epigenetic targets and tailoring epigenetic modifier therapy to achieve optimal gene derepression may represent a new therapeutic modality for OS. This analysis suggests that such a strategy may be applicable to a majority of osteosarcoma cases.

## MATERIALS AND METHODS

### Cell lines, tissue samples, and tumor samples

The human osteosarcoma cell lines HOS (TE85), MNNG/HOS cl 5, U-2 OS, G-292 cl A141B1, Saos-2, and MG-63 were obtained from the American Tissue Culture Collection (ATCC) and were propagated in Eagle Minimal Essential Medium supplemented with 10% fetal bovine serum (EMEM/10%FBS) at 37°C in a humidified 5% CO_2_ incubator.

Primary or metastatic human OS tumor samples were obtained from initial patient biopsies or tumor resections performed at Akron Children’s Hospital (Akron, OH, USA) and Rainbow Babies and Children’s Hospital (Cleveland, OH, USA). Additional human osteosarcoma samples were obtained from the Cooperative Human Tissue Network (CHTN, Columbus, OH, USA). Normal human bone genomic DNA was prepared from surgical specimens from excision of supranumerary digits in accordance with Akron children’s Hospital IRB regulations. Human growth plate genomic DNA was kindly provided by Dr. William Landis (University of Akron, USA). Sections of mouse distal femur growth plate tissue were kindly provided by Dr. Walter Horton, Northeast Ohio Medical University, and were prepared in accordance with Association for Assessment and Accreditation of Laboratory Animal Care accreditation requirements. Sections of human tissues including anterior pituitary, infant rib-end growth plate, and human capital femoral physis were obtained from archived tissue specimens in the Akron Children’s Hospital Department of Pathology. Acquisition of all human tissue samples was regulated by IRB-approved study protocols.

### Preparation of genomic DNA

High molecular weight genomic DNA was prepared from fresh or flash-frozen issue samples. Tumor specimens were finely minced or pulverized and DNA was extracted from tumor fragments or powder by SDS/proteinase K-digestion and phenol/chloroform extraction. For cultured cell lines, genomic DNA was purified from lysed cells using the 5PRIME ArchivePure DNA Blood kit (ThermoFisher, Waltham, MA, USA) according to the manufacturer’s directions.

### Southern blot analysis

Southern blot analysis for NNAT methylation was performed as described previously [14]. Blots were hybridized with the ^32^P-labeled 1.6 kb upstream *NNAT* fragment to identify a 1.6 kb digestion fragment representing alleles lacking methylation a the NruI site within the *NNAT* CpG island versus a 6Kb fragment representing methylated alleles (Figure 1). The proportion of methylated alleles was quantitated for each sample by densitometric analysis of the autoradiograph under visible light utilizing an EC3 imaging system with a digital CCD camera supported by VisionWorks LS 6.0 software (UVP, Upland, CA, USA).

### Combined Bisulfite Restriction Assay (COBRA)

One microgram of genomic DNA was subjected to bisulfite modification using the EpiTect Fast Bisulfite Conversion kit (Qiagen, Waltham, MA, USA). Modified DNA was subjected to PCR amplification (94° × 40 sec; 52.2° × 40 sec; 72° × 40 sec, 43 cycles) with primers F-AATCTTTATTCCCTAACAAAC and R-GGGTGGGATAGGGTTTTTAATT, which are specific to modified *NNAT* sequences 1530–1551 and 1716–1738, respectively (Accession U31767). To the 208 bp *NNAT* amplification product 500 ng of a 779 bp human *cMYC* PCR fragment was then added as a Taq^α^1 digestion control. The *cMYC* fragment was amplified from unmodified human DNA (Accession L00057; bases 361-1140; F-CTACGGAGGAGCAGCAGAGAAAG, R-AGAAGCGGGTCCTGGCAGCGG). Samples were digested with Taq^α^1. To control for completeness of bisulfite modification, duplicate reactions were digested with ApoI, since modification creates an ApoI site at *NNAT* position 1563. Digested samples were fractionated in polyacrylamide gels and visualized by UV illumination following ethidium bromide staining. Band intensity was quantitated by digital imaging as described above. The proportion of methylated *NNAT* alleles was calculated as 81 bp + 127 bp bands/81 bp + 127 bp + 208 bp bands. Only samples exhibiting complete (≥ 95%) modification and digestion were analyzed.

### Bisulfite sequencing

One microgram of bisulfite-modified genomic DNA from OS cell lines was subjected to 35 cycles of PCR amplification with the bisulfite modification-specific *NNAT* CpG island primers as above. Amplification products were cloned into the vector pCR2.1 (ThermoFisher, Waltham, MA, USA). At least 10 independent clones from each PCR reaction were submitted for sequencing. The prevalence of methylation in genomic DNA was determined at each of the 24 CpG sites within the amplimer from the proportion of cloned alleles exhibiting modification to TG (unmethylated) versus preservation of CG (methylated).

### RT-PCR

Total RNA was prepared from cultured cells with the 5PRIME PerfectPure RNA Blood kit (ThermoFisher, Waltham, MA, USA) and was reverse transcribed (SuperScript First Strand cDNA synthesis kit, ThermoFisher, Waltham, MA, USA). End point PCR was performed as described previously [14] using primers which amplify both *NNAT* mRNA isoforms *NNATα* (262 bp) and *NNATβ* (181 bp). Duplicate reactions were amplified with primers for the ubiquitously-expressed *SNRPD3* gene (500 bp). DNA was electrophoretically separated with a 2% Agarose gel with gel red and detected under ultraviolet light.

### Immunoblot

Whole cell protein was extracted from cultured cells following lysis in Radio-Immunoprecipitation Assay (RIPA) buffer with protease inhibitor. Ten microgram aliquots were separated on NuPage 10% Bis-Tris 1.5 mm gels (ThermoFisher, Waltham, MA, USA) and transferred to polyvinylidene difluoride (PVDF) membrane. Membranes were incubated overnight with primary antibody and then goat anti-rabbit-HRP (Santa Cruz Biotech, Dallas, TX, USA; sc-2004, 1:10000) for an hour followed by detection by enhanced chemiluminescence (ECL). Primary antibodies included NNAT (Abcam, Cambridge, MA, USA; ab27266, 1:1000), β-actin (Santa Cruz Biotechnology, Dallas, TX, USA).

### 5-aza-2′-deoxycytidine (5aza-dC) treatment

U-2 OS and MNNG/HOS OS cells were treated with freshly-prepared 5 mM 5aza-dC (Sigma-Aldrich, St. Louis, MO, USA). DNA and RNA were extracted from treated and untreated cells at 72 hours and evaluated by COBRA for *NNAT* promoter region methylation status and RT-PCR for expression of *NNAT* mRNA.

### Immunohistochemisty for NNAT

Sections of formalin fixed, paraffin embedded normal tissue or primary OS samples were subjected to heat based epitope retrieval, and peroxide blocking was performed according to standard protocols. 5% goat serum (sc-2043) and 1% bovine serum albumin (BSA) (Sigma) was used for blocking. Slides were then incubated over night with NNAT antibody (ab27266, 1:250) and 1% BSA as negative control. HRP conjugated secondary antibody (ab6721, 1:500) was used for detection with chromogen (Dako, Agilent Technologies, Santa Clara, CA, USA). Slides were counterstained with hematoxylin and analyzed by light microscopy.

### OS cell line transfections

The NNATα and NNATβ coding sequences (262 bp) were amplified by RT PCR from total RNA extracted from the NNAT expressor OS cell line HOS (TE-85) using primers (F: CCA ACA GCG GAC TCC GAG ACC AG and R: GTG TAT GCC AGC TTC TGC AGG GAG). PCR products were cloned into the expression vector pcDNA 3.1Neo (ThermoFisher, Waltham, MA, USA). The integrity of wild-type *NNAT* sequences encoded by the expression vectors was confirmed by DNA sequencing. OS cell lines were transfected with linearized vector using the FuGENE 6 transfection reagent (Promega, Madison, WI, USA), and colonies were grown under G418 selection (400 μM) for clonogenicity assays or for isolation of stable NNAT-expressor cell lines. For stable cell lines, macroscopic colonies were selected, expanded, and screened by immunoblot analysis for NNAT expression. To generate stable, inducible NNAT expression in OS cell lines, the LacSwitch II system (Agilent Technologies) was utilized according to the manufacturer’s direction. The NNATα or NNATβ cDNA was subcloned into the vector pORSVI/MCS to generate expression vectors. Cells were then sequentially transfected with the vector pCMVLacI followed by pORSVIHNNA, pORSVIHNNB, or pORSVI/MCS. Stable cell lines were isolated under the antibiotic selection appropriate for each transfection. Cell lines exhibiting negligible NNAT expression at baseline with robust NNAT expression upon treatment with isopropyl β-D-1-thiogalactopyranoside (*IPTG*) were utilized for experiments.

### Clonogenicity assays

Following transfection, cells were seeded into 100 mm dishes at uniform density and subjected to G418 selection. When colonies were macroscopically visible, cells were fixed and stained on the plate with methylene blue. Colonies were counted by visual inspection. Experiments were repeated three times.

### Cell proliferation and thapsigargin cytotoxicity assays

Cells were seeded at 5000 cells per well into 96 well tissue culture plates in complete medium without selective antibiotic. Cell numbers were then measured daily employing metabolic activity as a surrogate for viable cell number, using the CellTiter 96 Assay (Promega, Madison, WI, USA) according to the manufacturer’s instructions. For cytotoxicity assays, cells were cultured in 96 well plates overnight and then treated with thapsigargin (Sigma-Aldrich) or the DMSO-based dilution vehicle. Cell counts were determined as above.

### *In vitro* invasion assay

Twenty four-well plate cell culture inserts with 8.0 μm pores size (BD BioSciences, Franklin Lakes, NJ, USA) were coated with 50 μl of 0.5 mg/ml Matrigel^®^ matrix solution (BD Biosciences). Serum-containing media without selective antibiotic was placed in the lower chambers of wells, and 200 μl of cell suspension at 5 × 10^4^ cells/ml in serum-free media without selective antibiotics was dispensed onto the matrix coated inserts. Cells were incubated for 72 hours at 37°C in 5% CO_2._ After removing noninvading cells from upper chamber with a cotton swab, invading cells were fixed with 10% formalin. After inserts were stained with hematoxylin, cells were counted visually by light microscopy. Four inserts were examined for each cell line per experiment. The experiment was repeated three times.

### Measurement of intracellular calcium concentration ([Ca^2+^]_i_)

Cells were incubated (37°C, 5% CO_2_) for 45 min with fura-2 acetoxy methylester (fura-2/AM; 2 μM) in their respective media. Cover slips containing the fura-2-loaded cells were placed in a chamber (Warner Instruments, Hamden, CT, USA) mounted on the stage of an Olympus IX-81 inverted fluorescence microscope (Olympus America, Lake Success, NY, USA). The cells were superfused continuously with Hepes-buffered saline (HBSS) containing the following: 118 mM NaCl, 4.8 mM KCl, 1.2 mM MgCl_2_, 1.25 mM CaCl_2_, 11.0 mM dextrose, 5 mM pyruvate and 25 mM HEPES at a flow rate of 2 ml/min. The cells were primarily exposed to ATP for 2 mins, followed by a switch back to HBSS buffer for washout. Measurements of intracellular calcium [Ca^2+^]_i_ were performed on individual cells using a fluorescence imaging system (Easy Ratio Pro, Photon Technology International, Lawrenceville, NJ, USA) equipped with a multi wavelength spectrofluorometer (DeltaRAM X) and a QuantEM 512SC electron multiplying CCD camera (Photometrics, Tuscon, AZ, USA). Representative images and photometric data were acquired by alternating excitation wavelengths between 340 and 380 nm (20 Hz) and monitoring an emission wavelength of 510 nm. The ratio of the light intensities at the two wavelengths was used to measure changes in [Ca^2+^]_i_.

### Statistical analysis

All data were analyzed using GraphPad Prism software. For cell culture experiments, a one-way ANOVA or *t*-test were used based on the experiment. A Tukey post-hoc test was used for multiple comparisons. All data are represented as mean plus the standard error of the mean (SEM). Each experiment was repeated 3–6 times with three-four replicates per experiment.

## SUPPLEMENTARY MATERIALS


