# Supplementary material for: Aberrant epigenetic silencing of neuronatin is a frequent event in human osteosarcoma
Source: Oncotarget. 2020 May 19;11(20):1876–93. doi: 10.18632/oncotarget.27583 (PMC7244018; doi:10.18632/oncotarget.27583)
Supplement: Supplementary file 1 [file oncotarget-11-1876-s001.pdf]

Aberrant epigenetic silencing of neuronatin is a frequent event in human osteosarcoma

SUPPLEMENTARY MATERIALS

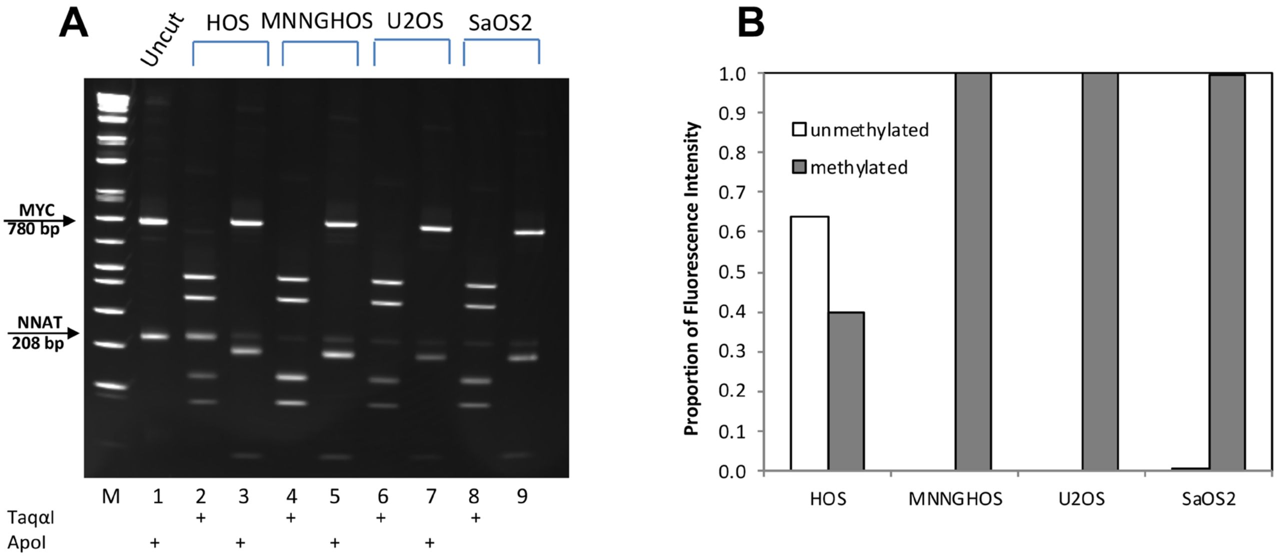

Supplementary Figure 1: COBRA analysis demonstrates *NNAT* hypermethylation in osteosarcoma cell lines. (**A**) COBRA analysis was performed on bisulfite-modified cell line DNA as described. PCR products were digested with Taq$^{\alpha}$I or ApoI. A 280 bp *MYC* CpG island PCR product was added to each *NNAT* amplification product as a control for completeness of Taq$^{\alpha}$I digestion. Digested DNA was fractionated on a polyacrylamide gel. Restriction fragment lengths are as follows: *MYC*/Taq$^{\alpha}$I – undigested 780 bp, digested – 424 bp and 356 bp; *NNAT*/Taq$^{\alpha}$I – undigested 208 bp, digested 127 bp and 81 bp. DNA samples are (lanes L to R): M, 1 kb ladder; 1, undigested control PCR products; 2, HOS/Taq$^{\alpha}$I; 3, HOS/ApoI; 4, MNNGHOS/Taq$^{\alpha}$I; 5, MNNGHOS/ApoI; 6, U-2 OS/Taq$^{\alpha}$I; 7, U-2 OS/ApoI; 8, Saos-2/Taq$^{\alpha}$I; 9, Saos-2/ApoI. (**B**) The proportions of methylated versus unmethylated *NNAT* alleles were quantitated for Figure 2A by analysis of the ethidium bromide-stained gel under UV illumination as described. Shown is a representative result of 3 experiments.

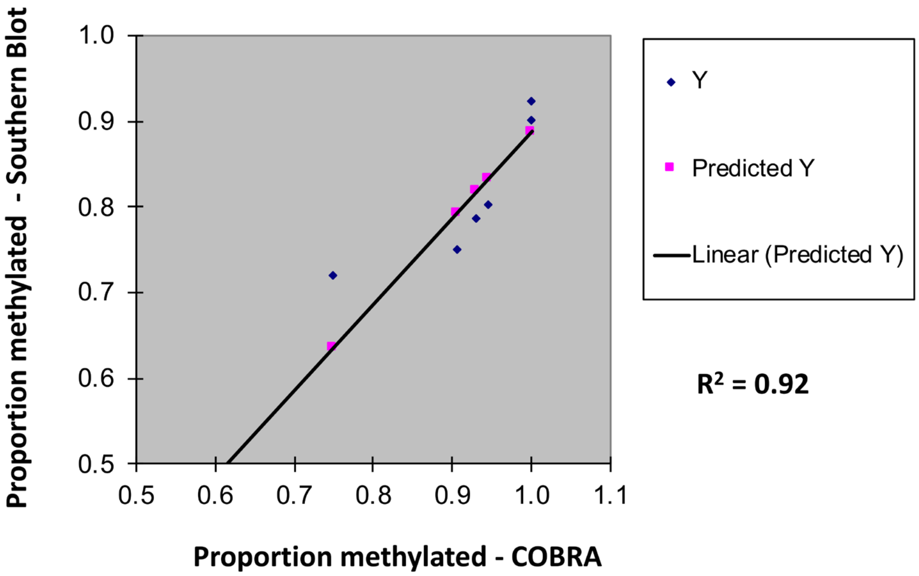

Supplementary Figure 2: Allelic methylation as quantitated by Southern blot analysis versus COBRA was compared for 7 osteosarcoma tumor samples by regression analysis. Shown are the proportions of methylated alleles detected by each modality. An R^2 of 0.92 was observed.

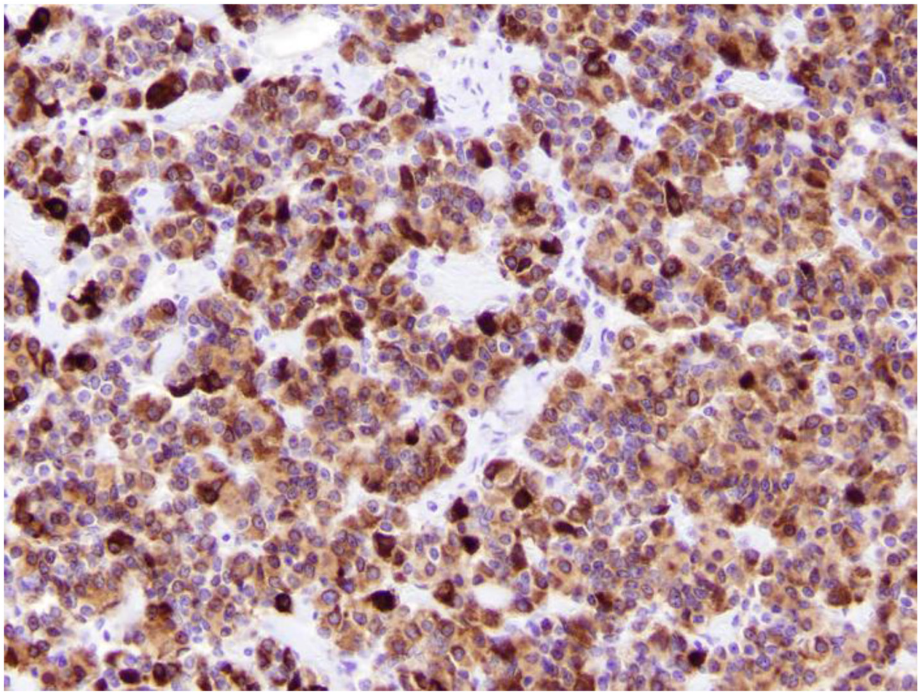

Supplementary Figure 3: Neuronatin expression was detectable in human anterior pituitary cells. A slide of human anterior pituitary tissue was treated with an antibody to human neuronatin and secondary antibody.

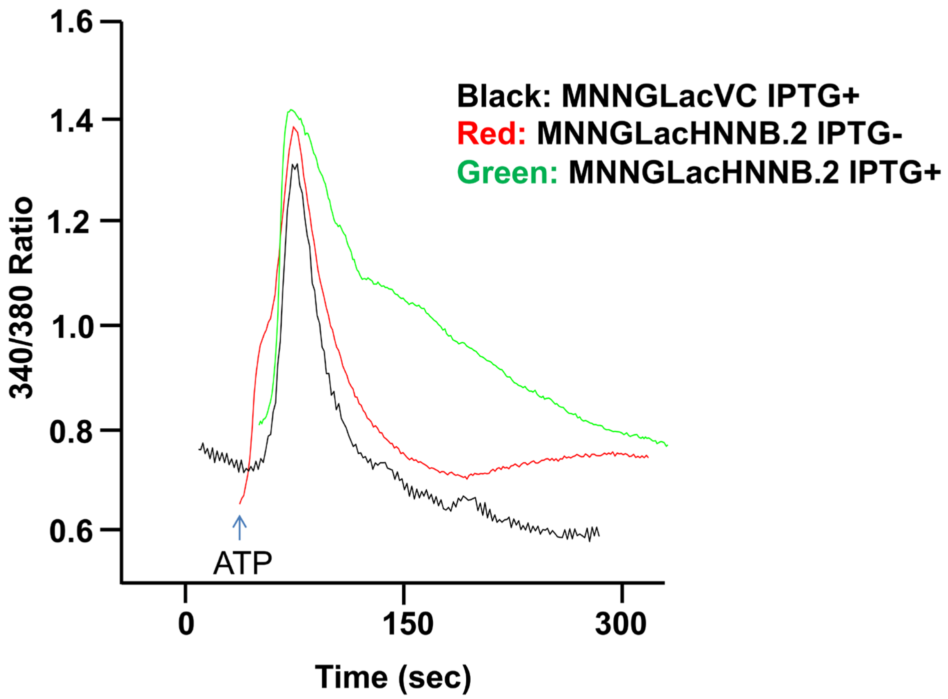

Supplementary Figure 4: $[Ca^{2+}]_i$ was measured by fluorescence imaging in fura-2/AM-loaded cells following ATP stimulation. HNNB cells were imaged with and without IPTG induction of NNATβ expression. VC cells are vector-control non-expressors. Shown are representative tracings from 3 replicate experiments.
